# Valorization of Fruit By-Products Through Lactic Acid Fermentation for Innovative Beverage Formulation: Microbiological and Physiochemical Effects

**DOI:** 10.3390/foods13233715

**Published:** 2024-11-21

**Authors:** Elisabetta Chiarini, Valentina Alessandria, Davide Buzzanca, Manuela Giordano, Negin Seif Zadeh, Francesco Mancuso, Giuseppe Zeppa

**Affiliations:** Department of Agricultural, Forest and Food Sciences (DISAFA), University of Turin, 10095 Grugliasco, TO, Italy; elisabetta.chiarini@unito.it (E.C.); valentina.alessandria@unito.it (V.A.); manuela.giordano@unito.it (M.G.); negin.seifzadeh@unito.it (N.S.Z.); francesco.mancuso786@edu.unito.it (F.M.); giuseppe.zeppa@unito.it (G.Z.)

**Keywords:** lactic acid bacteria, functional food, bioactive compounds, gas chromatography, volatile compounds

## Abstract

The increase in food production is accompanied by an increase in waste, particularly agricultural by-products from cultivation and processing. These residues are referred to as agricultural by-products. To address this issue, biotechnological processes can be used to create new applications for these by-products. This study explored the use of LAB strains (*Lactiplantibacillus plantarum, Streptococcus thermophilus*, *Lactobacillus delbrueckii* subsp. *bulgaricus*, and *Limosilactobacillus fermentum*) on by-products such as white grape pomace, cocoa bean shells, apple pomace, and defatted roasted hazelnut to develop yoghurt-style fruit beverages. Microbial load and pH changes were monitored during a 24 h fermentation and 14-day shelf life at 5 °C. Concentrations of sugars, organic acids, and volatile organic compounds were also analyzed using HPLC and GC-qMS. The results showed that optimizing the matrix led to significant bacterial growth, with viable microbes remaining under refrigeration. In particular, the strain of *L. plantarum* tested on the cocoa bean shell yielded the most promising results. After 24 h of fermentation, the strain reached a charge of 9.3 Log CFU/mL, acidifying the substrate to 3.9 and producing 19.00 g/100 g of lactic acid. Aromatic compounds were produced in all trials, without off-flavours, and characteristic fermented food flavours developed. Additionally, secondary metabolites produced by lactic acid bacteria may enhance the health benefits of these beverages.

## 1. Introduction

Population growth has led to a corresponding increase in the food industry and production [1]. A corollary to the increase in food production is an increase in waste; as reported by the United Nations Environment Programme, about 1.05 billion tonnes of food went to waste in 2022 [2].

Within the entire food production process, a significant percentage of the waste is generated from fruit and vegetable production. From the field to harvest, post-harvest, retail and consumption, around 45% of the total quantity produced is lost in the production chain, generating substantial waste [1,3].

Residues from the cultivation and processing of agricultural products that can be used as subsequent objects and not only as waste are called agricultural by-products. They are largely generated due to the misuse of fruits and vegetables, which tend to be seasonally overproduced and are primarily wasted during the preparation process [3,4]. In the case of vegetables, the main by-products are peels, seeds, stems and leaves, while for fruits, they are peels, skins and seeds [4].

The main challenge in this regard is the reuse of by-products to enhance their positive natural properties. It has been shown that the by-products of plant food processing contain significant amounts of valuable compounds that could be recovered and used as biologically active components or as natural food ingredients [5]. These could replace synthetic additives, thereby attracting considerable interest from both the industry and the scientific community [6]. In particular, high molecular weight components of the plant cell wall, such as pectin in apple peel [6], as well as secondary plant metabolites such as the phenolic compounds, carotenoids and components of the plant defence system, have antioxidant, antimicrobial and UV protection properties [5].

By-products can also directly be reused in food industries as ingredients to obtain functional products [4]. For instance, grape pomace, a by-product of winemaking, contains high levels of fibre and bioactive compounds and has been used as a replacement for white flour to make biscuits with significant antioxidant activity [7] but also incorporated into yoghurts to obtain a fortified product [8].

Considering these aspects, it is interesting to exploit biotechnological processes to define new applications of by-products. Very interesting is the fermentation process, whereby technological microorganisms can exploit the fruit or vegetable substrate to conduct metabolic reactions to obtain functional fermented products with high added value [1]. For example, considering the by-products formed during orange processing, orange peels were fermented with lactic acid bacteria such as *Lactobacillus casei, Lactobacillus plantarum* and *Lactobacillus paracasei* to obtain a solid state of fermentation [9].

Due to their high content of carbohydrates, polyphenols, vitamins, minerals, and fibre, fruits and vegetables are excellent starting matrices for lactic acid fermentation. Several studies have focused on producing fruit and vegetable-based fermented juices using lactic acid bacteria [10]. It has been shown that apricot juice is an excellent substrate for the growth of probiotic bacteria precisely because of the intrinsic characteristics of the matrix and that fermentation of the juice by microorganisms of the genus *Bifidobacterium* and *Lactobacillus* increases the antioxidant activity of the finished product [11].

Another aspect to consider is the growing interest in vegan or vegetarian diets and lactose intolerance [10,12]. In these circumstances, plant-based functional drinks with and without probiotic bacteria may be an attractive option for dairy-free consumers [13]. Microorganisms capable of lactic fermentation in fruit and vegetables make it possible to obtain functional foods with an aroma profile that positively influences the consumer’s final choice [14]. For instance, *Lactobacillus acidophilus*, *Lactobacillus rhamnosus*, *L. casei,* and *L. plantarum* were used to ferment apple juice to enrich the aromatic profile of the final product [14].

Considering the above-mentioned aspects, this study focused on applying lactic fermentation with different LAB strains (*Lactiplantibacillus plantarum*, *Streptococcus thermophilus*, *Lactobacillus delbrueckii* subsp. *bulgaricus* and *Limosilactobacillus fermentum*) on fruit by-products, such as the white grape pomace of cv *Moscato* (MP), cocoa bean shells (CBSs), apple pomace (AP) and de-fatted roasted hazelnut (DH) used directly as substrates to obtain for the first time to our knowledge a fruit beverage in yoghurt-style. These by-products were chosen for their richness in bioactive compounds and fibre, and several studies have already evaluated their application as ingredients in foods and microbial growth; sugars, organic acids and volatile compounds were evaluated during the production phase and refrigerated storage. The difference between this research and other research in this area is that the by-product of flour was used as the main ingredient of the proposed drink. This aspect makes the product highly sustainable from a circular economy perspective. Furthermore, as a completely plant-based product, it can be appreciated by consumers who follow a vegetarian diet and are placed in a market where food trends are increasingly linked to less meat consumption.

## 2. Materials and Methods

### 2.1. Lactic Acid Bacteria

Six lactic acid bacteria (LAB) strains were used for the experiments. Three were commercial LABs (Sacco S.r.l, Milan, Italy): *L. plantarum* Lyofast LPAL, *L. plantarum* Lyoflora V3 and the consortium of *S. thermophilus* and *L. delbrueckii* subsp. *bulgaricus* Y450B. The other three were non-industrial LABs from the Department of Agricultural, Forest and Food Sciences (DISAFA) collection: an *L. plantarum* S2T10D filed in the Turin University Culture Collection (TUCC) TUCC00000017 [15], and two strains of *Limosilactobacillus fermentum* (A1_02 and A1_14) isolated from cocoa bean fermentations [16].

The lyophilized cultures were stored at −25 °C, while non-industrial strains were stored in a solution with 30% glycerol (G5516, Sigma-Aldrich, St. Louis, MI, USA) at −80 °C. The strains were revitalized from cryogenics or lyophilized freeze-culture by resuspending the solutions in De Man, Rogosa and Sharpe (MRS) broth (CM0359, Oxoid, Basingstoke, United Kingdom) and incubating them at 37 °C for 24 h. Single colonies were obtained on MRS agar (CM0361, Oxoid, Basingstoke, United Kingdom) and incubated for 48 h at 37 °C under microaerophilic conditions (AG0026A, Oxoid, Basingstoke, United Kingdom). *S. thermophilus* and *L. delbrueckii* subsp. *bulgaricus* were isolated, respectively, on M17 agar and MRS agar (CM0785B and CM0785, Oxoid, Basingstoke, United Kingdom) from the lyophilized consortium (incubation at 48 h at 37 °C under microaerophilic conditions). The bacterial inoculation for the fermentations was prepared from a 24 h broth incubation at 37 °C to reach 9 Log CFU/mL. After centrifugation to discard the media, the cells were resuspended in Ringer’s solution (115525, Merck, Darmstadt, Germany). The suspension was then inoculated into the matrix to achieve a concentration of 7 Log CFU/mL (1% *v*/*v*).

### 2.2. By-Products

Four by-products were selected for this work: white grape pomace from the *Moscato* cultivar (MP), cocoa bean shells (CBSs), apple pomace (AP) and de-fatted flour from roasted hazelnut (DH). MP and CBS are furnished by local industries, while AP and DH were supplied as flours by Dohler (Darmstadt, Germany) and Pariani SRL (Givoletto, Turin, Italy), respectively.

The fresh MPs were dehydrated at 75 °C to obtain a humidity lower than 13%, and then were grounded and sieved (<1000 μm) with an Ultra-centrifugal Mill ZM 200 (Retsch GmbH, Haan, Germany). Similarly, the CBS were grounded and sieved (<1000 μm) with the Ultra-centrifugal Mill ZM 200 (Retsch GmbH, Haan, Germany).

The nutritional composition of the by-products used is reported in Supplementary Table S3.

### 2.3. Optimization of Fermentation Conditions

For the MP, CBS, and AP flours, it was necessary to raise the pH to 5.0 to ensure the better fermentation of the microorganisms. In the case of the CBS flour, 4% *w*/*w* D-glucose was added, as there were no fermentable sugars in the CBS. Initial trials with higher concentrations of flour (>20%) yielded negative results, possibly due to the high content of antibacterial compounds, such as polyphenols, in the raw matrix [17]. The case was different for the DH flour, which proved to be an excellent starting point for the fermentation of lactic acid bacteria without modifying the acidity and the sugar content of the solution.

Based on the results of these preliminary tests, a 15% *w*/*w* was used for the MP and CBS flours. The pH was adjusted to 5 using calcium bicarbonate, and for CBS, 4% *w*/*w* D-glucose (G8270; Sigma-Aldrich, St. Louis, MI, USA) was added. A 10% *w*/*w* suspension was used for AP and DH flours. For AP, the pH was adjusted to 5 with calcium bicarbonate, and for the S2T10D strain used in the AP beverage, 4.5% D-glucose was added.

### 2.4. Fermentation Process

For the beverage fermentation, all the flours were mixed with natural mineral water (Sant’Anna, Cuneo, Italy), homogenized using a BAGMIXER 400 for two minutes (Interscience, Saint-Nom-la-Bretèche, France), transferred into a flask, and pasteurized at 70 °C for 1 h in a thermostatic bath TW-12 JULABO (Labortechnik, Seelbach, Germany).

The fermentation process, performed in aerobic conditions, was monitored before microbial inoculation (T0), after 4 (T4), 7 (T7) and 24 (T24) hours. After 24 h, the beverages were stored at 5 °C and two additional sampling points were taken at 7 (D7) and 14 days (D14) to assess the stability of the microbial population. Each test was conducted in duplicate, and each measurement was taken in triplicate. For each sampling point, microbiological counts were performed, and the pH was measured using a Five Easy F20 pH-metre (Mettler Toledo, Greifensee, Switzerland). Gas chromatography–quadrupole mass spectrometry (GC-qMS) and HPLC analyses were performed on the T0 and T24 samples.

### 2.5. Microbiological Evaluation

At each sampling point, 1 mL of the solution was collected and used for enumeration by serial decimal dilutions with Ringer’s solution (115525, Merck, Darmstadt, Germany) to track the bacterial population trend on selective media. After pasteurization, the total aerobic count on Plate Count Agar—PCA (84608, VWR, Radnor, PA, USA)—was monitored to confirm the abatement of the flour’s original microbial population. MRS agar (CM0359, Oxoid, Basingstoke, United Kingdom) was used for *Lactoplantibacillus* spp., *Lactobacillus* and *Limosilactobacillus* strains, while M17 agar (CM0785B, Oxoid, Basingstoke, United Kingdom) was used to assess the growth of *S. thermophilus*. The plates were then incubated at 37 °C for 48 h. Violet Red Bile Glucose (VRBG) agar (CM0485, Oxoid, Basingstoke, UK) was used at each sampling point to detect possible contamination by the enterobacteria population, with plates incubated at 37 °C for 24 h. The blend’s pH values were determined at each sampling point using a basic 20 pH-metre Five Easy F20 (Mettler Toledo, Greifensee, Switzerland).

### 2.6. Sugars and Organic Acids Analysis

The sugars and organic acid concentrations were evaluated at T0 and T24 with an HPLC-DAD/RI system. The samples were centrifuged for 10 min (6000× *g*) at 10 °C, and the supernatant was filtered through a 0.20 µm disposable syringe membrane filter (Sartorius AG, Göttingen, Germany). The HPLC system (Thermo Electron Corporation, Waltham, MA, USA) was equipped with an SCM 100 degasser, an isocratic pump (P2000), a multiple autosampler (AS3000) fitted with a 20 µL loop, and a UV detector (UV100) set at 210 and 290 nm and a refractive index detector (RI-150). The detectors were connected in series. Data were collected on an EZChrom ver. 6.6 system (Thermo Electron Corporation, Waltham, MA, USA). The analyses were performed isocratically at 0.6 mL/min and 65 °C with a 300 × 7.8 mm i.d. cation exchange column Aminex HPX87H (Bio-Rad Laboratories, Hercules, CA, USA) equipped with a Cation H^+^ Microguard cartridge (Bio-Rad Laboratories, Hercules, CA, USA). The mobile phase was 0.013 N H_2_SO_4_ prepared by diluting reagent-grade sulfuric acid with distilled water, filtered through a 0.45 µm membrane filter (Sartorius, AG, Göttingen, Germany) and degassed under a vacuum. Each sample was analyzed three times. Analytical grade reagents were used as standards (Sigma-Aldrich Corporation, Milano, Italy).

### 2.7. Volatile Organic Compounds Analysis

The volatile organic compounds (VOCs) were evaluated at T0 and T24. The VOCs were extracted through headspace solid phase microextraction (HS-SPME) and analyzed, identified and quantified with gas chromatography coupled with a quadruple spectrometer (GC-qMS).

For the VOCs’ extraction, 0.5 g of the matrix was weighted in a 20 mL vial, and 10 µL of 1,3,5-tris(1-methylethyl) benzene (94.4 ppm) was added as the internal standard (IS). The vials were immediately capped with a PTFE–silicon septum. The extraction was performed using an AOC-5000 CombiPAL Autosampler for SPME (CT Analytics AG, Zwingen, Switzerland) equipped with an HS-SPME unit. Samples were conditioned at 60 °C for 15 min.

SPME fibre coated with divinylbenzene/carboxen/polydimethylsiloxane (Supelco, Belfonte, PA, USA) was then exposed to the headspace of the sample at 60 °C for 30′. The fibre was then desorbed in the injection port of the GC system in split mode at 260 °C.

GC-qMS analyses were performed on a Shimadzu GC-2010 gas chromatograph with a quadruple spectrometer (Shimadzu Corporation, Kyoto, Japan). The VOCs were separated on an RTX-5 capillary column (20 m length; 0.10 mm diameter and 0.10 µm film thickness). The oven time temperature was programmed as follows: 40 °C for 1 min, from 40 °C to 130 °C at the rate of 5 °C/min, then to 250 °C at 4 °C/min, and then to 300 °C at 27 °C/min rate. The final temperature was then held for 5 min. The used carrier was helium at a 0.65 mL/min constant flow with the split injection mode (1:15). The MS fragmentation was performed by the electronic ionization (EI) mode (70 eV), and the temperature of the ion source and quadrupole was 200 °C. The data were recorded in full-scan mode in the mass acquisition range of 33–300 with 0.30 s of scan time [18]. Two replicates were performed for each sampling point.

The identification of the volatile organic compounds was performed by comparing the EI-MS fragmentation pattern of each compound with those available on the National Institute of Standards and Technology (NIST17) mass-spectral library and chemical standards. The semi-quantitative concentrations of the VOCs identified were calculated as the area of each volatile component divided by the response factor of the added internal standard and expressed as micrograms of internal standard equivalents per kg of the sample (μg IS eq./kg of sample). Data were acquired and analyzed using GC-MS Solution Workstation software (version 4.3) (GC-MS Solution, Shimadzu Corporation, Kyoto, Japan).

### 2.8. Statistical Analysis

Statistical analyses were performed using RStudio 2022.07.2 (R version 4.2.1) software. Data uniformity was evaluated with Shapiro–Wilk’s W and Modified Levene’s tests (Brown–Forsythe test). The ANOVA test was used to evaluate differences between multiple groups, and Dunn’s corrected Bonferroni test was used for non-parametric data. For post hoc analysis, Tukey’s test was used. Multivariate analysis was made using R’s package “vegan” version 2.6-8 to obtain information regarding the different strains considering the different molecules identified by HPLC and GC-qMS. Graphs were produced with ggplot2 version 3.4.1.

## 3. Results

### 3.1. Dynamics of LAB Loads and pH

Due to the difference in the physicochemical characteristics of by-products, preliminary tests were conducted to determine the optimal values for each by-product in terms of the flour percentage in the beverage, initial pH of the beverage, and sugar concentration in the starting suspension. After the pasteurization, the microbial load was below 1 Log CFU/mL for enterobacteria and below 2 Log CFU/mL for the aerobic count. All LABs were inoculated in the beverages at 7.0 Log CFU/mL.

**Figure 1:**
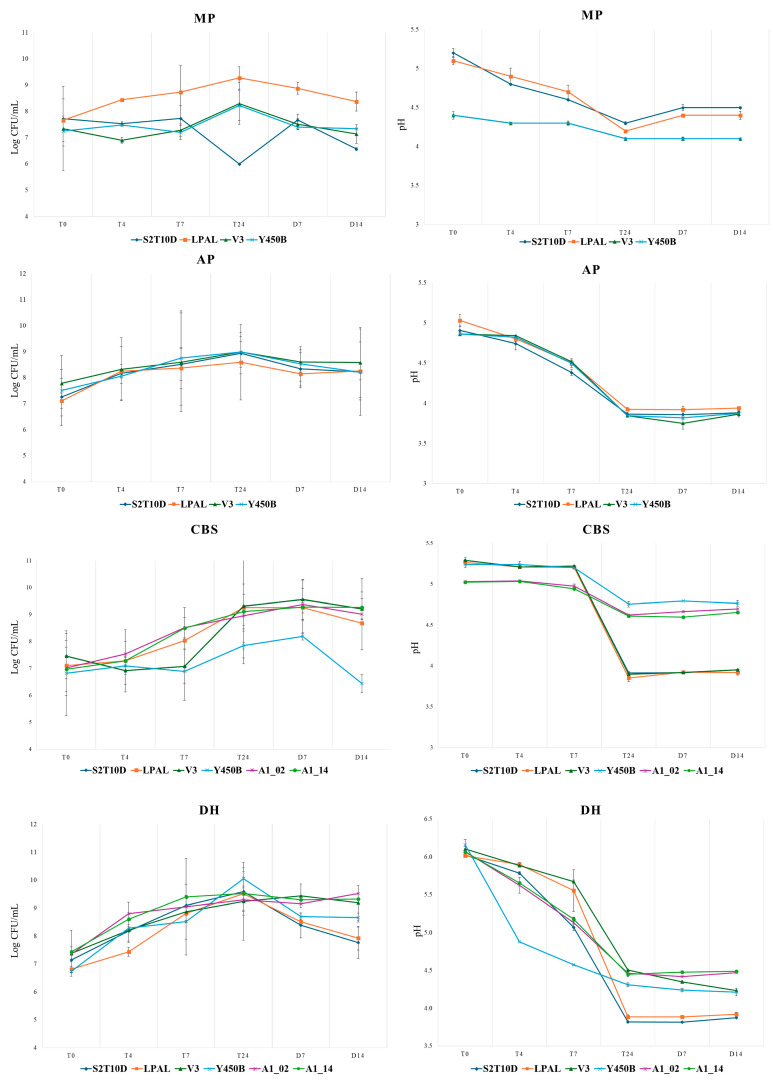
Evolution of the bacteria count (Log CFU/mL; **left**) and pH (**right**) of the LAB strains in *Moscato* grape pomace (MP), cocoa bean shells (CBSs), apple pomace (AP) and de-fatted roasted hazelnut (DH) during 24 h of incubation at 37 °C and 14 days at 5 °C. Bars represent statistical variances in triplicate measurements of duplicate fermentations (Dunn’s test, Bonferroni, *p* > 0.025).

For the MP beverage (Figure 1 and Supplementary Table S1), the *L. plantarum* LPAL and the V3 showed an increasing trend during the fermentation, reaching the highest load after 24 h of incubation (respectively 9.3 and 8.3 Log CFU/mL), while at the same sampling time, S2T10D decreased its load to 6.0 Log CFU/mL. Regarding the consortium Y450B, *S. thermophilus* maintained a constant load during the sampling points T4 and T7, then resumed growth during the 24 h incubation period and reached a load of 8.2 Log CFU/mL, with the same value recorded for *L. delbrueckii* subsp. *bulgaricus.* During shelf life (D7 and D14), the bacterial load remained constant for LPAL, V3 strains and Y450B consortium, while it decreased for S2T10D. The higher bacterial load achieved by the LPAL and V3 strains after 24 h of fermentation also led to higher acidification of the matrix, reaching the lowest pHs of 4.2 and 4.1, respectively.

For the AP beverage (Supplementary Table S1), *L. plantarum* strains showed an increasing trend in the first 24 h of fermentation, reaching their highest load. After 24 h of fermentation, S2T10D reached a load of 8.9 Log CFU/mL, LPAL of 8.6 Log CFU/mL and finally, V3 reached 9.0 Log CFU/mL. Similarly, in the case of the Y450B consortium, the highest bacterial load was observed at 24 h of fermentation, reaching a load of 9.1 Log CFU/mL for *L. delbrueckii* subsp. *bulgaricus* and 8.8 Log CFU/mL for *S. thermophilus.* During the storage period at refrigerated temperatures, the bacterial load slowly decreased over 24 h but remained constant for all strains at around 8.0 Log CFU/mL. The AP matrix subjected to fermentation by all strains showed a decrease in pH to 3.8 (Figure 1).

*L. plantarum* strains inoculated on the cocoa bean shell (CBS) by-product reached a load of 9.3 Log CFU/mL after 24 h of fermentation (Figure 1, Supplementary Table S1). In the first 7 h of fermentation, the growth of LPAL was constant, while for the V3 and S2T10D strains, the load dropped to 6.9 Log CFU/mL after 4 h and then increased to 7.1 Log CFU/mL at the T7 sampling point. The Y450B consortium on the CBS matrix reached a load of 7.9 Log CFU/mL (value obtained by averaging the loads of the two strains) after 24 h of fermentation. While the strain *S. thermophilus* remained constant during the incubation period, reaching a load of 6.6 Log CFU/mL, the strain *L. delbrueckii* subsp. *bulgaricus* showed a decrease from T4 to T7 from 7.2 to 6.9 Log CFU/mL and reached a load of 8.1 Log CFU/mL at T24. The CBS substrate was then subjected to fermentation with strains A1_02 and A1_14, both of which were *L. fermentum*. In this case, both strains showed similar fermentative power, reaching a load of around 9.0 Log CFU/mL after 24 h of fermentation, and they exhibited constant growth in the first few hours of incubation (Figure 1, Supplementary Table S1). The fermented CBS beverage during the *shelf-life* maintained a high bacterial load with both *L. plantarum* and *L. fermentum* strains, whereas the bacterial count recorded dropped after fourteen days (D14) of storage following fermentation with Y450B (6.4 Log CFU/mL) (Figure 1, Supplementary Table S1). The pH dynamics shown in Figure 1 indicate the similar acidification of the substrate in S2T10D, LPAL and V3 trials, with an initial value of 5.3 that decreased after 24 h to 3.9. On the other hand, less evident acidification was measured following the inoculation of the strains Y450B, A1_02 and A1_14, with respective pH values at T24 of 4.8, 4.6, and 4.6 (Figure 1, Supplementary Table S1).

Finally, in the case of DH flour fermented with the S2T10D, LPAL, and V3 strains, a similar growth trend was also shown, reaching the highest load after 24 h. In particular, for S2T10D, the load was 9.6 log CFU/mL; for LPAL, it was 9.5 Log CFU/mL; and for V3, it was 9.2 Log CFU/mL (Figure 1, Supplementary Table S1). The fermentation conducted by the Y450B lactic acid bacteria exhibited the highest bacterial count achieved on the DH matrix compared to all fermentation tests. After 24 h of incubation, it reached a higher mean count with the greater presence of *S. thermophilus* compared to *L. delbrueckii* subsp. *bulgaricus* (Figure 1, Supplementary Table S1). As observed with the CBS beverage, the *L. fermentum* strains exhibited a similar trend when fermenting DH flour, showing consistent growth in the initial hours of fermentation and reaching the highest load after 24 h. Specifically, A1_02 reached a load of 9.3 log CFU/mL, while A1_14 reached 9.5 Log CFU/mL. During the refrigeration period, the count decreased to around 7.8 log CFU/mL (D14) for beverages fermented with strains S2T10D and LPAL (Figure 1, Supplementary Table S1). Similarly, for the Y450B consortium, the bacterial count decreased after seven days of storage, reaching the final charge of 8.7 Log CFU/mL. However, this trend was different for the bacterial load of strains V3, A1_02 and A1_14 even after 14 days; the recorded counts were approximately 9.2 Log CFU/mL for strain V3 and for both *L. fermentum* strains. Regarding the acidification of the DH beverage, the lowest pH value was measured with S2T10D and LAPL strains at the sampling point T24 (3.8 and 3.9, respectively). The pH value reached 4.5 in fermentations with V3, A1_02, and A1_14 after 24 h (Figure 1, Supplementary Table S1).

### 3.2. Sugar and Organic Acids Composition

The results of organic acids and sugar HPLC evaluation on MP, AP, DH, and CBS before inoculation and after 24 h of fermentation are presented in Supplementary Table S2.

**Figure 2:**
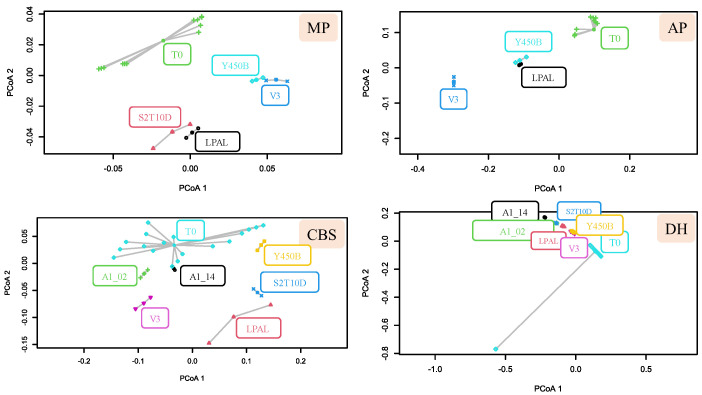
PCoA on data dispersion produced on the multivariate distance matrix from sugars and organic acids detected by HPLC analysis. For each fruit by-product, the results obtained for each strain are reported. For each matrix, the initial condition (T0) was compared to the fermented beverage after 24 h.

In MP, ten compounds were identified and quantified. In V3 and Y450B trials, D-malic acid exhibited a lower concentration compared to the initial time point (−0.81 g/100 g and −0.69 g/100 g, respectively). Lactic acid production was observed in all MP trials, with the LPAL fermentation yielding the highest amount, resulting in a lactic acid concentration 6.0 times higher than at T0. Acetic acid in MP was statistically lower in V3 trials compared with T0 (−0.13 g/100 g). A decrease in isobutyric acid concentration was observed in V3 and Y450B, with a decrease of 1.08 g/100 g and 1.01 g/100 g, respectively. The amount of glucose in MP was statistically different only in V3 trials compared to T0, showing a decrease of 27.22%. Uric acid, tartaric acid, and fructose remained constant concerning the time zero. The PCoA analysis applied to sugars and organic acids in MP (Figure 2) demonstrated a clear differentiation between the fermented matrix and the raw matrix: Y450B and V3 were grouped as S2T10D and LPAL.

The HPLC analysis on CBS beverages revealed a significant reduction in citric acid concentrations after fermentation with LPAL (−2.37 g/100 g), V3 (−0.95 g/100 g) and A1_02 (−1.05 g/100 g) strains. Similarly, the tartaric acid concentrations decreased after fermentations with V3 (−1.04 g/100 g) and A1_02 (−0.74 g/100 g). Regarding lactic acid, no significant differences were observed between the raw CBS beverage (T0) and fermentation with *L. fermentum* and V3 strains, while fermentations with S2T10D and LPAL showed a 7.73-fold increase. No significant differences were found in any fermentations tested for malic acid. The acetic acid and pyroglutamic concentrations increased only in the CBS fermented with Y450B with an increase from T0 0.23 to 0.35 g/100 g and from T0 1.42 to 3.69 g/100 g, respectively. Glucose was consumed in every CBS trial. Significant changes in glucose concentration were observed in the LPAL, V3 and Y450B fermentation, with glucose levels decreasing from 36.82 g/100 g at T0 to 29.40, 28.81, and 26.83 g/100 g, respectively. Principal Coordinates Analysis (PCoA) applied to the CBS data revealed distinct groupings based on the fermentation trials. The S2T10D and LPAL trials clustered together, while the *L. fermentum* strains did not show significant differences from the raw CBS beverage, as depicted in Figure 2.

Regarding the AP fermentation, citric and pyruvic acid concentrations did not change during fermentation. Lactic acid and acetic acid were produced by every strain, though in varying amounts. The highest production was observed in V3 trials, with concentrations of lactic and acetic acid, respectively, at 3.90 and 0.17 g/100 g. Glucose was consumed in every trial, while fructose was consumed only by the V3 strain (−89.57%). The AP data regarding S2T10D trials are not shown and compared to the others due to the addition of 4.5% D-glucose to the matrix (Supplementary Table S2). In the S2T10D trials, glucose was consumed, and lactic acid was produced in amounts like in the other trials (Supplementary Table S2). The PCoA on AP data shows the differentiation in the fermented product from the starting one, with the clusterization of the strains LPAL and Y450B (Figure 2).

Citric acid, pyruvic acid, and raffinose concentrations in DH did not show significant changes during the fermentations (Supplementary Table S2). The succinic acid concentration decreased in every trial (average decrease of 47.5%), except for fermentations with *L. fermentum* strains, where no significant change was observed. The D-malic acid concentration decreased in all trials (average decrease of 34.66%). The lactic acid amount increased in every trial (average 17.33-fold increase), with statistical similarities between LPAL and S2T10D (average 21.64-fold increase). An average increase of 4.74-fold in the acetic acid concentration was observed in all forms of fermentation, reaching the maximum value of 5.95 g/100 g in V3 trials. The isobutyric acid concentration increased from an average of 0.18 g/100 g to an average of 0.39 g/100 g. Glucose was consumed in higher quantities from LPAL, S2T10D, A1_02 and A1_14 strains. The glucose and fructose consumption were assessed in every trial with a lower entity in V3 fermentation. Ethanol was only produced by both *L. fermentum* strains (1.13 g/100 g). The PCoA plot does not show a clear separation between the fermented substrates and the raw flour (Figure 2).

### 3.3. Volatile Organic Compounds Content

**Figure 3:**
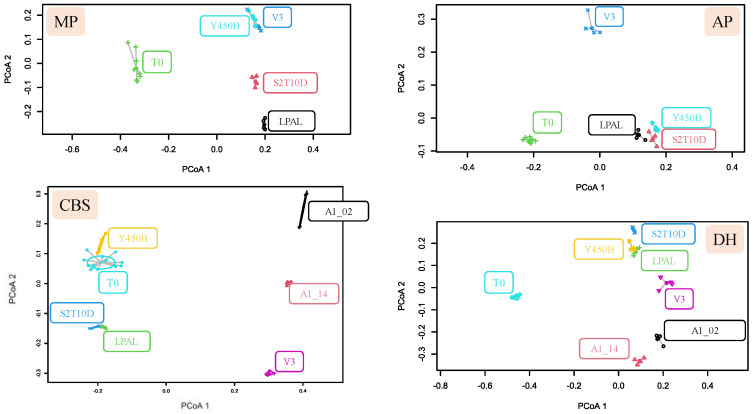
PCoA on data dispersion produced on the multivariate distance matrix from VOCs. For each fruit by-product, the results obtained for each strain are reported. For each matrix, the initial condition (T0) is compared to the fermented beverage after 24 h.

**Figure 4:**
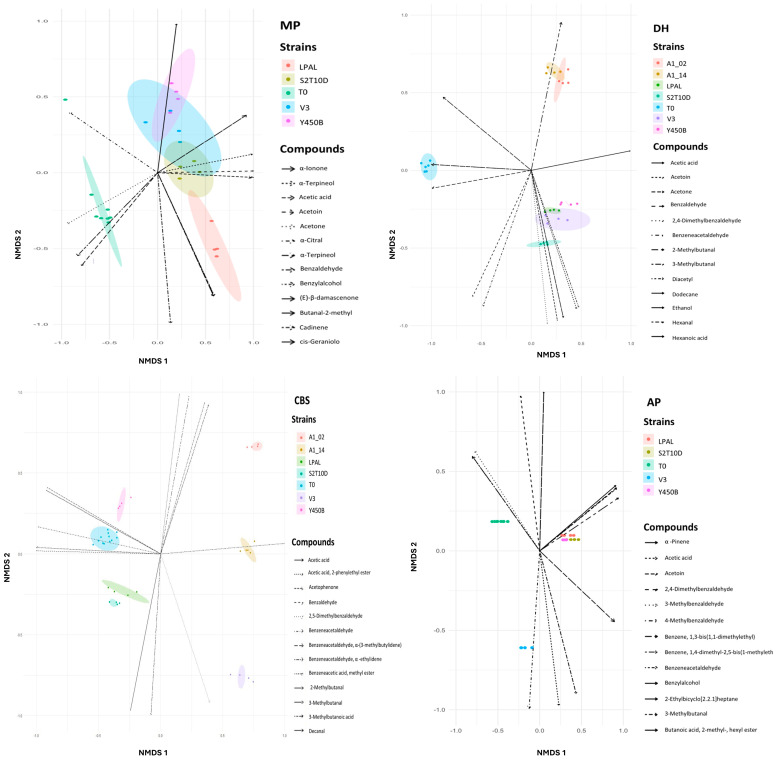
Non-metric multidimensional scaling (NMDS) on data dispersion on VOCs. For each fruit by-product, the results obtained for each strain are reported. The main volatile compounds produced during each fermentation are highlighted.

The 128 volatile compounds identified (Supplementary Table S4) in MP at T0 and T24 were clustered in 11 classes (Table 1, Figure 3). The acid content increased significantly during all the fermentations (Table 1), with acetic acid being the predominant acid. Alcohol compounds were generated in all trials, particularly in LPAL fermentation. The Y450B consortium primarily consumed aldehyde compounds. Ester compounds were produced by all the strains except Y450B with the highest values observed in LPAL fermentation. Regarding the furan derivate compound group, dihydro-5-pentyl-2(3H)-furanone was produced by LPAL (Supplementary Table S4). Hydrocarbon compounds were consumed by all strains except LPAL, for which they remained stable. The ketone compounds concentration increased after 24 h in both the LPAL and V3 trials. γ-Nonalactone was the only lactone produced in fermented beverages inoculated with S2T10D, V3 and Y450B strains (Supplementary Table S4). Phenol derivate compounds were produced by all strains, with a higher amount in the fermentation with LPAL. Terpene compounds increased in LPAL fermentation. PCoA was applied to the VOCs (Figure 3) and demonstrated that the fermented solution differentiated from the raw material. Y450B and V3 fermentation were grouped in the same zone of the plot. Non-metric multidimensional scaling (NMDS) was also applied to the VOCs, revealing the main molecules that influenced the differentiation between the sample before the fermentation and after 24 h by the strains (Figure 4).

A total of 91 compounds from CBS (Supplementary Table S4) were identified and clustered into ten classes (Table 1). Acids, mainly represented by acetic acid, were produced by LPAL and S2T10D. Alcohols were produced as well, except in the fermentation by Y450B (Supplementary Table S4). Aldehydes were produced by LPAL, S2T10D, and Y450B. Esters were higher in LPAL trials, with an abundance of pentatonic acid ethyl esters. Ketone compounds presented higher values in LPAL, S2T10D, and Y450B fermentations. Lactone compounds were produced by the two *L. fermentum* strains and V3. Phenol derivate compounds were consumed in *L. fermentum* and V3 trials, while in Y450B, an increased concentration was assessed due to the formation of benzaldehyde. Terpene concentrations increased after fermentation with V3 and *L. fermentum* strains. PCoA was used to visualize the data of the different fermented products and showed a similarity between the matrix before fermentation and Y450B components. *L. plantarum* strains S2T10D and LPAL were grouped in the same zone of the plot (Figure 3). NMSD (Figure 4) was also performed to identify the main molecules that differentiated the trials.

Ten classes (Table 1) divided into 137 compounds were identified from AP fermentations (Supplementary Table S4). Overall, acids were consumed by *L. plantarum* LPAL and S2T10D. In every trial, nonanoic acid was consumed, but in V3- and Y450B-fermented substrates, acetic acid and octanoic acid were produced. Alcohols were produced by all strains in similar quantities, while aldehydes, benzene, and furan derivatives remained stable in every trial. The Y450B and S2T10D trials showed a decrease and an increase in terpene concentrations, respectively. Esters were produced by S2T10D and consumed by V3. Hydrocarbon concentrations increased only in S2T10D and Y450B trials. Ketones were produced by every strain except by V3. PCoA in Figure 3 was performed on the data and showed similarities between S2T10D, LPAL, and Y450B trials grouped in the same area of the plot, while V3 differentiated from both this group and T0. NMSD analysis (Figure 4) was performed and highlighted the main compounds that participated in the differentiation of the samples.

A total of 59 compounds (Supplementary Table S4) were identified and grouped into nine classes from the DH tests (Table 1). Acids were produced by all strains except in the A1_14 fermentation (Figure 4). An increase in alcohol concentration was observed in every fermentation except for Y450B. A decrease in aldehyde concentration was observed during all trials except for S2T10D trials. Esters and benzene derivatives concentrations increased only in S2T10D trials. Hydrocarbons were consumed in every trial. Ketones were produced by LPAL, S2T10D, Y450B, and A1_02 due to the production of 2-octen-4-one, and they remained stable in the V3 and A1_14 trials. Terpene concentrations were higher in the fermentation with S2T10D.

PCoA analysis (Figure 3) showed S2T10D, LPAL, Y450B, and V3 trials grouped in the same area of the plain while *L. fermentum* trials differentiated from both this group and T0. Finally, the NMSD (Figure 4) was also performed to identify the main molecules that differentiated the trials. It shows how the different molecules produced led to the differentiation of the strains used in the fermentations. It is evident that the strains during the 24 h fermentation time separated from the starting matrix as a result of the production of volatile compounds.

## 4. Discussion

This study involved the use of fruit by-product flours to develop a new fermented beverage obtained without milk and with a high number of lactic bacteria. The strain Lyofast LPAL is commonly recommended in the production of dairy products, while V3 is specially indicated for fermented vegetable products. The *L. plantarum* TUCC00000017 (S2T10D) was chosen since it was isolated from table olives, is characterized as a probiotic and is used for the production of Toma Piemontese PDO [15].

In most of the tests performed, *L. plantarum* strains, both industrial and non-industrial, reached the highest bacterial load after 24 h of fermentation (Figure 1, Supplementary Table S1) and remained viable for up to 14 days of refrigeration. For the MP beverage, the LPAL strain achieved the highest bacterial load during fermentation, which was also maintained during shelf-life.

The S2T10D strain is the only one to have reached the lowest count after 24 h on MP flour. For the DH flour, the S2T10D led to the highest acidification within 24 h of fermentation. Utilizing *L. plantarum* species to create functional products has already yielded promising results with several fruit matrices [10,13]. The industrial microbial consortium Y450B consists of the species *S. thermophilus* and *L. delbrueckii* subsp. *bulgaricus* was optimized for yoghurt production and selected to produce exopolysaccharides. Y450B growth in MP and CBS beverages was not positive, even after the optimization of the beverage, especially in the first hours of the fermentation. On the contrary, this microbial consortium inoculated on AP flour shows high growth within 24 h of fermentation, resulting in the acidification of the substrate. The most promising results were obtained with DH flour, where the use of Y450B as a starter culture resulted in high lactic acid bacteria loads that remained stable even during cold storage.

The results obtained show that, with the optimization of the matrix, it is possible to obtain a high bacterial growth with maximum loads ranging from 8 to 10 Log CFU/mL, depending on the inoculated strains. It is also interesting to note that the microbial load remains viable even when the product is kept at refrigeration temperature. Chemical and microbiological analysis point out that by-products with high concentrations of polyphenols, such as CBS and MP, can be of great interest due to the positive effect of these molecules on human health [19,20], but they can also have significant problems with fermentation according to the antimicrobial activity of these compounds [21] then it is necessary to change the pH and sugar concentration.

The HPLC analysis showed the significant production of lactic acid and consumption of glucose and fructose for all the beverages, with differences depending on the strain used. The highest lactic acid production was recorded for the *L. plantarum* species, but particularly interesting are the results obtained with LPAL and S2T10D on the MP, DH, and CBS beverages. According to Figure 2, for the fermented MP beverage, the LPAL and S2T10D strains differentiated in the same area of the plot, highlighting that the composition of sugars and organic acids in MPs changes significantly after fermentation and that these changes depend on the LAB used. The grouping patterns suggest similarities in the metabolic profiles of different strains (Y450B and V3, S2T10D and LPAL) post-fermentation. However, in the case of the fermented product obtained from AP, the greatest production of lactic acid was from the fermentation with the V3 strain. In all fermented products, glucose was consumed, with a considerable reduction compared to the initial product (T0). In particular, the strains that consumed the most were V3 and the Y450B consortium in MP and CBS. On the other hand, the consumption of fructose in the AP fermented with the V3 strain after 24 h of fermentation was evident. These data can be associated with studies that assess the isolation and identification of different strains from fructose-rich environments, where fructophilic lactic acid bacteria are denominated [22,23]. In the case of the DH beverage, glucose and fructose are consumed mainly by strains A1_02, A1_14 and LPAL. Comparing the different fermented by-products, the change in citric acid during fermentation was evident only in the CBS trials. Citric acid was metabolized by LPAL, V3 and A1_02, indicating the activation of the metabolic pathway of citrate by these strains, leading to the formation of both acetoin and diacetyl in different trials [24,25]. These results confirm what has already been shown in various works, whereby the metabolism of carbohydrates with different LAB species varies according to strain and substrate type [26]. As expected, glucose was the main molecule consumed [27]. Given the use of LAB, it is not surprising that the main acid produced was lactic acid, resulting from lactic fermentation [28]. The concentration of this molecule not only contributes to the acidification of the product but also has an antimicrobial action [29].

The analysis of the volatile compounds showed that in all trials, aromatic compounds were produced, and no off-flavour production occurred. The graphs in Figure 3 and Figure 4 show that, in most cases, there is a production of aromatic compounds that leads to the differentiation of the fermented product from the starting sample. The PCoA analysis conducted on the profile of organic acids and sugars (Figure 2) highlights the formation of clusters between the strains, which is confirmed by the same analysis conducted on the profile of volatile compounds (Figure 3).

In the fermented product obtained on CBS and MP flour, the highest acid production was found with the LPAL and S2T10D strains, the production of which is practically absent in the case of the AP fermented product. DH fermentation resulted in higher acid production following fermentation with strain *L. fermentum* A1_02.

The NMSD graph (Figure 4) illustrates how the different strains are grouped into distinct clusters due to the presence of different molecules. The separation of the T0 sample from all fermentations is evident. Interesting to note how the *L. fermentum* strains clustered in the fermentation of DH flour compared to the production of benzaldehyde. Benzaldehyde production and its derivates differentiate the aroma profile of MP, DH, and CBS, promoting a typical almond scent [30]. Acetoin is one of the main compounds produced in the fermentation of MP, DH, and AP, and it is known to contribute to sweet, buttery, creamy and dairy flavours [31]. Diacetyl, present in the DH fermentation, usually contributes to the butter-like aroma in different dairy products after fermentation [32].

## 5. Conclusions

The application of lactic acid bacteria to non-dairy products is becoming increasingly interesting, particularly given the topic of the growing interest in plant-based diets among the population. Furthermore, the reuse of by-products from industrial processing as a potential substrate for fermentation is an innovative aspect of the circular economy. This work highlights the different efficiencies of lactic acid bacteria inoculated as starters in by-product beverages to obtain promising results from innovative foods.

Typical flavour-giving molecules, characteristic of fermented foods, were produced during the production of these beverages, and the presence of different secondary metabolites produced by lactic acid bacteria can enhance the health-related aspects of fermented food. New studies are needed to optimize the beverage production while also considering the rheology aspects and, above all, the sensory aspects, which are very important for the consumer acceptability of the product. Moreover, the functional proprieties of these products can be further enhanced with the use of starters with attested probiotic capacities.

## Figures and Tables

**Table 1:** Average values (μg/kg) of different chemical classes pre-inoculum (T0) and after 24 h of fermentation in each strain trial (LPAL, S2T10D, V3, Y450B, A1_02 and A1_14). The mean between biological and analytical replicates is presented. Values with different letters are statistically different (Anova *p*-value < 0.05).

**MP**											
	Acids	Alcohols	Aldehydes	Benzofuran Derivatives	Esters	Furan Derivatives	Hydrocarbons	Ketones	Lactones	Phenol Derivatives	Terpenes
T0	2 ^c^	657 ^d^	1252 ^a^	12 ^a^	99 ^bc^	Not detected	23 ^a^	691 ^b^	Not detected	Not detected	1142 ^b^
LPAL	362 ^a^	1735 ^a^	973 ^ab^	Not detected	628 ^ab^	42 ^a^	23 ^a^	1398 ^a^	Not detected	208 ^a^	1974 ^a^
S2T10D	149 ^a^	1340 ^ab^	1023 ^ab^	Not detected	310 ^a^	2 ^b^	Not detected	405 ^b^	20 ^a^	37 ^bc^	1228 ^b^
V3	166 ^a^	1161 ^bc^	1296 ^a^	Not detected	302 ^c^	4 ^b^	Not detected	863 ^ab^	16 ^ab^	64 ^b^	1023 ^b^
Y450B	77 ^b^	724 ^cd^	481 ^b^	7 ^a^	78 ^d^	2 ^b^	Not detected	672 ^b^	8 ^bc^	47 ^bc^	812 ^b^
**CBS**											
	Acids	Alcohols	Aldehydes	Azotate derivatives	Esters	Furan derivative		Ketones	Lactones	Phenol derivatives	Terpenes
T0	534 ^b^	14 ^d^	126 ^c^	949 ^a^	114 ^bc^	Not detected		60 ^b^	Not detected	1023 ^b^	65 ^bc^
LPAL	1400 ^a^	268 ^bc^	234 ^b^	867 ^a^	251 ^a^	Not detected		212 ^a^	12 ^d^	770 ^bc^	39 ^c^
S2T10D	1250 ^a^	339 ^ab^	270 ^b^	982 ^a^	194 ^ab^	Not detected		243 ^a^	5 ^d^	641 ^bc^	37 ^c^
V3	787 ^b^	391 ^a^	0 ^d^	72 ^b^	124 ^bc^	51 ^a^		21 ^b^	605 ^b^	308 ^c^	157 ^a^
Y450B	512 ^b^	33 ^d^	487 ^a^	974 ^a^	131 ^bc^	Not detected		160 ^a^	Not detected	2061 ^a^	20 ^c^
A1_02	508 ^b^	246 ^c^	38 ^cd^	50 ^b^	70 ^c^	Not detected		4 ^b^	233 ^c^	131 ^c^	88 ^abc^
A1_14	590 ^b^	257 ^bc^	29 ^d^	178 ^b^	38 ^c^	Not detected		36 ^b^	951 ^a^	263 ^c^	126 ^ab^
**AP**											
	Acids	Alcohols	Aldehydes		Esters	Furan derivatives	Hydrocarbons	Ketones	Benzene derivatives	Phenol derivatives	Terpenes
T0	34 ^ab^	501 ^b^	4186 ^a^		263 ^bc^	58 ^a^	208 ^b^	228 ^b^	1984 ^a^	15 ^b^	189 ^ab^
LPAL	Not detected	1104 ^a^	4391 ^a^		333 ^ab^	44 ^a^	280 ^ab^	460 ^a^	2160 ^a^	20 ^ab^	185 ^ab^
S2T10D	6 ^c^	1204 ^a^	4426 ^a^		394 ^a^	41 ^a^	270 ^ab^	526 ^a^	2228 ^a^	23 ^ab^	310 ^a^
V3	24 ^bc^	1286 ^a^	3224 ^a^		88 ^d^	33 ^a^	337 ^a^	312 ^ab^	2242 ^a^	17 ^ab^	213 ^ab^
Y450B	62 ^a^	1042 ^a^	4374 ^a^		199 ^c^	41 ^a^	165 ^b^	530 ^a^	2078 ^a^	26 ^a^	96 ^b^
**DH**											
	Acids	Alcohols	Aldehydes	Azotate derivatives	Esters		Hydrocarbons	Ketones	Benzene derivatives		Terpenes
T0	Not detected	38 ^d^	288 ^a^	97 ^b^	Not detected		54 ^a^	81 ^c^	Not detected		114 ^bc^
LPAL	301 ^c^	320 ^c^	88 ^bc^	150 ^b^	Not detected		Not detected	239 ^b^	Not detected		157 ^b^
S2T10D	365 ^bc^	300 ^c^	234 ^a^	297 ^a^	22 ^a^		Not detected	423 ^a^	44 ^a^		220 ^a^
V3	539 ^b^	260 ^c^	53 ^c^	149 ^b^	Not detected		Not detected	69 ^c^	Not detected		65 ^c^
Y450B	189 ^cd^	46 ^d^	132 ^b^	0 ^c^	Not detected		Not detected	233 ^b^	Not detected		Not detected
A1_02	1290 ^a^	1133 ^a^	29 ^c^	144 ^b^	Not detected		Not detected	100 ^c^	Not detected		116 ^bc^
A1_14	10 ^d^	991 ^b^	11 ^c^	138 ^b^	Not detected		10 ^b^	62 ^c^	Not detected		98 ^bc^

## Data Availability

The original contributions presented in the study are included in the article/Supplementary Materials, further inquiries can be directed to the corresponding author.

## Supplementary Materials

The following supporting information can be downloaded at https://www.mdpi.com/article/10.3390/foods13233715/s1. Supplementary Table S1: Microbial loads and pH; Supplementary Table S2: HPLC data; Supplementary Table S3: Nutritional composition of the flour by-product; Supplementary Table S4: GC-qMS data.
