# Supplementary material for: Valorization of Fruit By-Products Through Lactic Acid Fermentation for Innovative Beverage Formulation: Microbiological and Physiochemical Effects
Source: Foods. 2024 Nov 21;13(23):3715. doi: 10.3390/foods13233715 (PMC11640541; doi:10.3390/foods13233715)
Supplement: Supplementary file 1 [file foods-13-03715-s001.zip › Supplementary tables 1_2_3.pdf]

Supplementary Table S1. For each condition, microbial loads (log CFU/ml), standard variations relative to microbial load, pH and standard deviation of pH measurements are reported. The variations of triplicate measurements of duplicate fermentation. The results for the Y450B consortium are the average of the values measured in the two member strains.

Moscato pomacee (MP)

LAB	Sampling time	Log10 CFU/mL	Dev.St Log CFU/mL	pH	Dev.St pH
S2T210D (TUCC00000017)	T0	7.7	0.58	5.2	0.1
S2T210D (TUCC00000017)	T4	7.5	0.02	4.8	0.0
S2T210D (TUCC00000017)	T7	7.7	0.15	4.6	0.0
S2T10D (TUCC00000017)	T24	6.0	0.00	4.3	0.0
S2T10D (TUCC00000017)	D7	7.7	1.24	4.5	0.0
S2T10D (TUCC00000017)	D14	6.6	0.41	4.5	0.0
LPAL	T0	7.7	0.07	5.1	0.0
LPAL	T4	8.4	0.54	4.9	0.1
LPAL	T7	8.7	0.04	4.7	0.1
LPAL	T24	9.3	0.17	4.2	0.0
LPAL	D7	8.9	0.34	4.4	0.0
LPAL	D14	8.4	0.24	4.4	0.1
V3	T0	7.3	0.01	4.4	0.0
V3	T4	6.9	0.13	4.3	0.0
V3	T7	7.3	0.27	4.3	0.0
V3	T24	8.3	0.07	4.1	0.0
V3	D7	7.5	0.29	4.1	0.0
V3	D14	7.1	0.20	4.1	0.0
Y450B	T0	7.3	0.05	4.4	0.0
S. thermophilus		7.2	0.07		
L. delbrueckii subsp. *bulgaricus*		7.3	0.14		
Y450B	T4	7.5	0.33	4.3	0.0
S. thermophilus		7.0	0.01		
L. delbrueckii subsp. *bulgaricus*		7.7	0.41		
Y450B	T7	7.2	0.10	4.3	0.0
S. thermophilus		7.0	0.08		
L. delbrueckii subsp. *bulgaricus*		7.4	0.10		
Y450B	T24	8.2	0.13	4.1	0.0
S. thermophilus		8.2	0.18		
L. delbrueckii subsp. *bulgaricus*		8.2	0.06		
Y450B	D7	7.4	0.42	4.1	0.0
S. thermophilus		6.9	0.11		
L. delbrueckii subsp. *bulgaricus*		7.6	0.49		
Y450B	D14	7.3	0.75	4.1	0.0
S. thermophilus		7.3	0.84		
L. delbrueckii subsp. *bulgaricus*		7.4	0.68		

Apple pomacee (AP)

LAB	Sampling time	Log10 CFU/ml	Dev.St Log CFU/mL	pH	Dev.St pH
S2T10D (TUCC00000017)	T0	7.3	0.08	4.9	0.1
S2T10D (TUCC00000017)	T4	8.2	0.04	4.7	0.1
S2T10D (TUCC00000017)	T7	8.5	0.11	4.4	0.0
S2T10D (TUCC00000017)	T24	8.9	0.07	3.9	0.0

S2T10D (TUCC00000017)	D7	8.3	0.08	3.9	0.1
S2T10D (TUCC00000017)	D14	8.2	0.22	3.9	0.0
LPAL	T0	7.1	0.23	5.0	0.1
LPAL	T4	8.3	0.25	4.8	0.0
LPAL	T7	8.4	0.07	4.5	0.0
LPAL	T24	8.6	0.02	3.9	0.0
LPAL	D7	8.2	0.15	3.9	0.0
LPAL	D14	8.3	0.03	3.9	0.0
V3	T0	7.8	0.13	4.9	0.0
V3	T4	8.3	0.03	4.8	0.0
V3	T7	8.6	0.01	4.5	0.0
V3	T24	9.0	0.11	3.8	0.0
V3	D7	8.6	0.19	3.8	0.1
V3	D14	8.6	0.02	3.9	0.0
Y450B	T0	7.5	0.05	4.9	0.0
S. thermophilus		7.5	0.99		
L. delbrueckii subsp. *bulgaricus*		7.5	0.39		
Y450B	T4	8.1	0.08	4.8	0.0
S. thermophilus		7.9	0.99		
L. delbrueckii subsp. *bulgaricus*		8.2	0.16		
Y450B	T7	8.8	0.21	4.5	0.1
S. thermophilus		8.8	0.98		
L. delbrueckii subsp. *bulgaricus*		8.8	0.18		
Y450B	T24	9.0	0.01	3.8	0.0
S. thermophilus		8.8	0.99		
L. delbrueckii subsp. *bulgaricus*		9.1	0.04		
Y450B	D7	8.5	0.17	3.8	0.0
S. thermophilus		8.5	1.01		
L. delbrueckii subsp. *bulgaricus*		8.6	0.18		
Y450B	D14	8.2	0.00	3.9	0.0
S. thermophilus		8.2	0.99		
L. delbrueckii subsp. *bulgaricus*		8.2	0.05		

Cocoa Bean Shell (CBS)

LAB	Sampling time	Log10 CFU/ml	Log Dev.St CFU/mL	pH	Dev.St pH
S2T10D (TUCC00000017)	T0	7.5	0.09	5.3	0.0
S2T10D (TUCC00000017)	T4	6.9	0.19	5.2	0.0
S2T10D (TUCC00000017)	T7	7.1	0.15	5.2	0.0
S2T10D (TUCC00000017)	T24	9.3	0.00	3.9	0.0
S2T10D (TUCC00000017)	D7	9.6	0.11	3.9	0.0
S2T10D (TUCC00000017)	D14	9.2	0.27	4.0	0.0
LPAL	T0	7.1	0.52	5.3	0.0
LPAL	T4	7.3	0.42	5.2	0.0
LPAL	T7	8.0	0.02	5.2	0.0
LPAL	T24	9.3	0.08	3.9	0.0
LPAL	D7	9.3	0.01	3.9	0.0
LPAL	D14	8.7	2.48	3.9	0.0
V3	T0	7.5	0.09	5.3	0.0
V3	T4	6.9	0.19	5.2	0.0
V3	T7	7.1	0.15	5.2	0.0
V3	T24	9.3	0.00	3.9	0.0

V3	D7	9.6	0.11	3.9	0.0
V3	D14	9.2	0.27	4.0	0.0
Y450B	T0	6.8	0.42	5.2	0.0
S. thermophilus		6.8	0.35		
L. delbrueckii subsp. *bulgaricus*		6.8	0.46		
Y450B	T4	7.1	0.10	5.2	0.0
S. thermophilus		6.9	0.42		
L. delbrueckii subsp. *bulgaricus*		7.2	0.24		
Y450B	T7	6.9	0.58	5.2	0.0
S. thermophilus		6.9	0.01		
L. delbrueckii subsp. *bulgaricus*		6.9	0.12		
	T24	7.9	0.42	4.8	0.0
S. thermophilus		6.6	0.12		
L. delbrueckii subsp. *bulgaricus*		8.1	0.34		
Y450B	D7	8.2	1.39	4.8	0.0
S. thermophilus		6.8	1.13		
L. delbrueckii subsp. *bulgaricus*		8.5	1.10		
Y450B	D14	6.4	0.01	4.8	0.0
S. thermophilus		6.3	0.21		
L. delbrueckii subsp. *bulgaricus*		6.6	0.24		
A1_02	T0	7.0	0.06	5.0	0.0
A1_02	T4	7.5	0.22	5.0	0.0
A1_02	T7	8.5	0.27	5.0	0.0
A1_02	T24	9.0	0.30	4.6	0.0
A1_02	D7	9.4	0.16	4.7	0.0
A1_02	D14	9.0	0.03	4.7	0.0
A1_14	T0	7.0	0.09	5.0	0.0
A1_14	T4	7.3	0.04	5.0	0.0
A1_14	T7	8.5	0.11	4.9	0.0
A1_14	T24	9.1	0.14	4.6	0.0
A1_14	D7	9.3	0.06	4.6	0.0
A1_14	D14	9.3	0.17	4.7	0.0

De-oiled hazelnut (DH)

LAB	Sampling time	Load Log10 CFU/ml	Log Dev.St CFU/mL	pH	Dev.St pH
S2T10D (TUCC00000017)	T0	7.1	0.00	6.0	0.0
S2T10D (TUCC00000017)	T4	8.2	0.19	5.8	0.0
S2T10D (TUCC00000017)	T7	9.1	0.20	5.1	0.0
S2T10D (TUCC00000017)	T24	9.6	0.06	3.8	0.0
S2T10D (TUCC00000017)	D7	8.4	0.16	3.8	0.0
S2T10D (TUCC00000017)	D14	7.8	0.12	3.9	0.0
LPAL	T0	6.8	0.38	6.0	0.0
LPAL	T4	7.4	0.33	5.9	0.0
LPAL	T7	8.8	0.47	5.6	0.3
LPAL	T24	9.5	0.11	3.9	0.0
LPAL	D7	8.5	0.39	3.9	0.0
LPAL	D14	7.9	0.17	3.9	0.0
V3	T0	7.4	0.07	6.1	0.0
V3	T4	8.2	0.16	5.9	0.0
V3	T7	8.9	0.05	5.7	0.0
V3	T24	9.2	0.02	4.5	0.0

V3	D7	9.4	0.11	4.4	0.0
V3	D14	9.2	0.10	4.2	0.0
Y450B	T0	6.7	0.06	6.2	0.1
S. thermophilus		7.0	0.13		
L. delbrueckii subsp. *bulgaricus*		6.2	0.39		
Y450B	T4	8.3	0.02	4.9	0.0
S. thermophilus		8.6	0.40		
L. delbrueckii subsp. *bulgaricus*		6.9	0.01		
Y450B	T7	8.5	0.10	4.6	0.0
S. thermophilus		8.8	0.12		
L. delbrueckii subsp. *bulgaricus*		7.5	0.39		
Y450B	T24	10.0	0.96	4.3	0.0
S. thermophilus		10.3	0.96		
L. delbrueckii subsp. *bulgaricus*		8.7	0.17		
Y450B	D7	8.7	0.20	4.2	0.0
S. thermophilus		8.9	0.11		
L. delbrueckii subsp. *bulgaricus*		8.3	1.17		
Y450B	D14	8.7	0.17	4.2	0.0
S. thermophilus		8.8	0.01		
L. delbrueckii subsp. *bulgaricus*		8.4	1.66		
A1_02	T0	7.3	0.25	6.1	0.0
A1_02	T4	8.8	0.18	5.6	0.1
A1_02	T7	9.1	0.01	5.1	0.1
A1_02	T24	9.3	0.17	4.5	0.0
A1_02	D7	9.2	0.00	4.4	0.0
A1_02	D14	9.5	0.00	4.5	0.0
A1_14	T0	7.4	0.11	6.1	0.0
A1_14	T4	8.6	0.01	5.7	0.0
A1_14	T7	9.4	0.04	5.2	0.0
A1_14	T24	9.5	0.11	4.5	0.0
A1_14	D7	9.3	0.30	4.5	0.0
A1_14	D14	9.3	0.04	4.5	0.0

Supplementary Table S2. Values (g/100 g) of sugars and organic acids were detected before inoculum and after 24h of incubation at 37°C. Data are represented ± standard deviation. Values with different letters are significantly different. (Anova. *p-value < 0.05*).

Moscato pomacee

Sampling point	Citric acid	Tartaric acid	D-malic acid	Lactic acid	Uric acid	Acetic acid	Isobutyric acid	Maltose	Glucose	Fructose
T0	6.12^{ab} (±0.481)	1.61^{a} (±0.759)	2.96^{ab} (±0.519)	1.23^{d} (±0.186)	0.03^{a} (±0.014)	0.94^{a} (±0.054)	2.15^{a} (±0.477)	0.52^{a} (±0.119)	18.11^{a} (±2.692)	18.10^{a} (±0.855)
S2T10D	6.49^{a} (±0.231)	1.04^{a} (±0.078)	3.45^{a} (±0.254)	5.64^{b} (±0.557)	0.02^{a} (±0.001)	0.94^{ab} (±0.045)	2.49^{a} (±0.198)	0.53^{a} (±0.202)	17.76^{ab} (±1.695)	18.02^{a} (±2.690)
LPAL	6.30^{ab} (±0.104)	0.99^{a} (±0.028)	3.17^{a} (±0.094)	7.27^{a} (±0.143)	0.02^{a} (±0.000)	0.95^{a} (±0.027)	1.67^{ab} (±0.054)	0.58^{a} (±0.024)	16.76^{ab} (±0.660)	18.29^{a} (±0.664)
V3	5.56^{b} (±0.256)	1.85^{a} (±0.100)	2.15^{c} (±0.051)	4.55^{c} (±0.135)	0.04^{a} (±0.002)	0.81^{b} (±0.020)	1.07^{b} (±0.069)	0.41^{a} (±0.028)	13.18^{b} (±0.924)	16.93^{a} (±1.160)
Y450B	5.45^{b} (±0.556)	1.89^{a} (±0.184)	2.27^{bc} (±0.179)	4.42^{c} (±0.440)	0.02^{a} (±0.025)	0.90 ^{ab} (±0.091)	1.14^{b} (±0.341)	0.44^{a} (±0.008)	14.62^{ab} (±0.350)	17.93^{a} (±0.490)

Cocoa Been Shell

	Citric acid	Tartaric acid	D-malic acid	Lactic acid	Pyroglutamic	Acetic acid		Maltose	Glucose	
T0	2.73^{ab} (±1.258)	1.72^{ab} (±0.623)	4.22^{b} (±3.375)	2.47^{c} (±2.085)	0.23^{bc} (±0.163)	1.42^{bc} (±1.201)		0.18^{a} (±0.020)	36.82^{a} (±3.617)	
S2T10D	3.30^{ab} (±0.092)	2.00^{ab} (±0.123)	6.36^{ab} (±0.096)	19.16^{a} (±1.139)	0.42^{ab} (±0.029)	3.05^{ab} (±0.063)		0.16^{ab} (±0.009)	31.87^{ab} (±1.888)	
LPAL	0.36^{b} (±0.010)	1.54^{ab} (±1.572)	3.94^{ab} (±3.050)	19.00^{a} (±1.748)	0.34^{abc} (±0.129)	2.69^{abc} (±1.350)		0.11^{bc} (±0.005)	29.40^{b} (±4.153)	
V3	1.78^{b} (±0.229)	0.68^{b} (±0.125)	0.25^{b} (±0.064)	5.13^{bc} (±0.595)	0.07^{c} (±0.009)	0.43^{c} (±0.068)		0.13^{bc} (±0.015)	28.81^{b} (±3.120)	
Y450B	4.36^{a} (±0.022)	2.52^{a} (±0.461)	10.07^{b} (±1.001)	8.92^{b} (±0.450)	0.35^{a} (±0.218)	3.69^{a} (±0.134)		0.16^{ab} (±0.075)	26.83^{b} (±3.622)	
A1_02	1.68^{b} (±0.100)	0.98^{b} (±0.088)	0.81^{b} (±0.269)	2.78^{c} (±0.073)	0.07^{bc} (±0.000)	0.90^{bc} (±0.024)		0.10^{c} (±0.006)	34.43^{ab} (±0.941)	
A1_14	2.20^{ab} (±0.053)	1.75^{ab} (±0.067)	2.92^{b} (±0.252)	3.41^{bc} (±0.013)	0.06^{bc} (±0.003)	0.80^{bc} (±0.071)		0.10^{c} (±0.001)	32.74^{ab} (±1.308)	

Apple pomacee

	Citric acid	Tartaric acid	D-malic acid	Lactic acid	Acetic acid	Fumaric acid	Pyruvic acid	Succinic acid	Glucose	Fructose
T0	0.22^{a} (±0.069)	0.02^{c} (±0.047)	0.72^{b} (±0.083)	0.04^{c} (±0.068)	0.01^{d} (±0.016)	0.01^{b} (±0.002)	0.01^{a} (±0.005)	0.01^{b} (±0.018)	5.01^{a} (±0.259)	9.49^{a} (±0.285)
LPAL	0.25^{a} (±0.021)	0.38^{a} (±0.029)	0.56^{b} (±0.023)	3.70^{b} (±0.120)	0.11b^{c} (±0.053)	0.01^{a} (±0.000)	0.01^{a} (±0.002)	0.00^{b} (±0.000)	2.80^{b} (±0.030)	9.25^{a} (±0.085)
V3	0.19^{a} (±0.096)	0.23^{ab} (±0.111)	1.08^{a} (±0.262)	3.90^{a} (±0.096)	0.17^{a} (±0.050)	0.00^{c} (±0.000)	0.01^{a} (±0.007)	0.04 ^{a} (±0.015)	2.62^{b} (±0.041)	0.99^{b} (±0.005)
Y450B	0.14^{a} (±0.036)	0.08b^{c} (±0.108)	0.65^{b} (±0.167)	3.68^{b} (±0.062)	0.13^{ab} (±0.001)	0.00b^{c} (±0.000)	0.05^{a} (±0.001)	0.02 ^{ab} (±0.003)	2.76^{b} (±0.458)	9.58^{a} (±0.418)
S2T10D (added 4.5% glucose)	0.24 (±0.18)	0.35 (±0.21)	0.65 (±0.25)	3.64 (±0.00)	0.07 (±0.01)	0.00 (±0.00)	0.02 (±0.02)	0.04 (±0.00)	43.04 (±1.00)	9.39 (±0.17)

De-oiled Hazelnut

	Citric acid	Raffinose	D-Malic acid	Lactic acid	Acetic acid	Isobutyric acid	Pyruvic acid	Succinic acid	Glucose	Fructose	Ethanol
T0	0.40^{a} (±0.053)	1.38^{a} (±0.079)	2.13^{a} (±0.229)	0.35^{d} (±0.304)	0.55^{d} (±0.494)	0.18^{c} (±0.108)	0.03^{ab} (±0.008)	0.10^{a} (±0.007)	3.23^{a} (±0.424)	1.28^{a} (±0.081)	0.00^{c} (±0.000)
LPAL	0.32^{a} (±0.026)	1.07^{a} (±0.075)	0.88^{bc} (±0.015)	7.82^{a} (±0.942)	1.49^{bc} (±0.323)	0.51^{a} (±0.110)	0.05^{a} (±0.007)	0.05^{c} (±0.005)	0.93^{bc} (±0.033)	0.54^{cd} (±0.056)	0.00^{c} (±0.000)
S2T10D	0.39^{a} (±0.161)	1.20^{a} (±0.217)	0.00^{c} (±0.000)	7.33^{a} (±0.164)	1.31^{cd} (±0.034)	0.41^{ab} (±0.017)	0.04^{ab} (±0.001)	0.05^{c} (±0.002)	0.84^{bc} (±0.002)	0.64^{bcd} (±0.015)	0.00^{c} (±0.000)
V3	0.31^{a} (±0.034)	1.22^{a} (±0.277)	1.44^{ab} (±0.251)	4.66^{c} (±1.090)	5.95^{a} (±1.071)	0.37^{abc} (±0.144)	0.04^{ab} (±0.006)	0.04^{c} (±0.003)	2.17^{ab} (±0.115)	1.24^{ab} (±0.277)	0.00^{c} (±0.000)
Y450B	0.46^{a} (±0.214)	1.37^{a} (±0.056)	1.23^{ab} (±0.160)	5.40^{bc} (±0.103)	2.01^{bc} (±0.444)	0.23^{bc} (±0.039)	0.02^{b} (±0.027)	0.05^{c} (±0.001)	1.94^{ab} (±0.331)	1.08^{abc} (±0.105)	0.00^{c} (±0.000)
A1_02	0.35^{a} (±0.001)	1.21^{a} (±0.000)	0.88^{bc} (±0.015)	5.54^{bc} (±0.025)	2.48^{b} (±0.118)	0.43^{ab} (±0.026)	0.05^{a} (±0.000)	0.15^{a} (±0.001)	0.10^{c} (±0.010)	0.28^{d} (±0.004)	1.08^{b} (±0.072)
A1_14	0.35^{a}	1.19^{a}	0.00^{c}	5.65^{b}	2.39^{bc}	0.42^{ab}	0.05^{a}	0.15^{a}	0.10^{c}	0.28^{d}	1.17^{a}

	(±0.004)	(±0.090)	(±0.000)	(±0.072)	(±0.052)	(±0.010)	(±0.001)	(±0.001)	(±0.002)	(±0.005)	(±0.036)

Supplementary Table S3. Nutritional composition of the flour by-product selected for the fermentation process.

Chemical parameters (g/100g)	MO	CBS	AP	DH
Protein	9.3	17.2	4.4	36.4
Fat	5.1	3.1	1.2	8.4
Carbohydrates	27.1	10.5	51.1	3.6
Moisture	5.7	5.2	8.6	4.2
Ash	4.5	6.8	1.7	5.4
TDF	48.1	56.7	32.8	41.6
